# The interplay between maternal employment dynamics and trends in breastfeeding practices over the past decade

**DOI:** 10.1186/s13052-024-01697-8

**Published:** 2024-08-09

**Authors:** Şeyma ÇİÇEK, Siddika Songül YALÇIN, Mehmet Ali ERYURT, Suzan YALÇIN

**Affiliations:** 1 Department of Social Pediatrics, Institute of Child Health, Hacettepe University, Ankara, Turkey; 2 Institute of Population Studies, Hacettepe University, Ankara, Turkey; 3 Department of Food Hygiene and Technology, Faculty of Veterinary Medicine, Selcuk University, Konya, Turkey; 4 Department of Pediatrics, Faculty of Medicine, Hacettepe University, Ankara, Turkey

**Keywords:** Breastfeeding, Working Mother, Demographic Health Survey, Trends

## Abstract

**Background:**

Working mothers are in a situation where they have to manage both their job and maternal roles simultaneously. We aim to show the relationship between mothers' breastfeeding behavior and working status, as well as the effect of their working conditions on the continuation of breastfeeding.

**Methods:**

This study examined 3557 (weighted 3490) child-mother pairs from Turkey's Demographic Health Survey data in 2008, 2013, and 2018 with a complex sample multiple logistic regression analysis to explore the relationship between breastfeeding and maternal employment.

**Results:**

In our study, 35.5% of the mothers had never worked, 18.6% were still working, and 45.9% had worked in the past but were not currently working. When breastfeeding percentages were examined based on maternal working status, mothers who worked in 2008 had significantly lower breastfeeding percentages than those who had never worked or had worked previously but not currently. In both 2013 and 2018, after legal regulations, maternal employment didn't affect breastfeeding. After adjusting for confounding factors, maternal employment in the industry sector had lower breastfeeding percentages [AOR:0.06 (95% CI: 0.01–0.48)] than those working in agriculture.

**Conclusion:**

It seems that adhering to legal regulations such as paid maternity leave and lactation leave can promote breastfeeding. It is necessary to raise awareness of mothers working in the industry about the continuation of breastfeeding and to monitor their legal rights.

**Supplementary Information:**

The online version contains supplementary material available at 10.1186/s13052-024-01697-8.

## Introduction

Breast milk is the ideal source of nutrients for optimal growth and development during infancy [1, 2]. Considering the countless benefits for the present and future of the baby-mother couple, encouraging breastfeeding is one of the most important preventive health approaches [3, 4]. For this reason, it is recommended to start breastfeeding within the first hour after birth, to feed babies exclusively with breast milk for the first 6 months, and to continue breastfeeding up to 2 years of age and beyond, with complementary feeding starting at 6 months [5]. Working women often have difficulties balancing the roles of mother and employee after giving birth; they are undecided between quitting their jobs and breastfeeding; in addition, working mothers experience insufficient milk anxiety after returning to work following childbirth, and breastfeeding time is reduced due to the lack of breastfeeding opportunities in their workplaces, so they tend to feed their babies formula. For this reason, they stop breastfeeding early [6, 7]. In a study conducted in Hong Kong, the breastfeeding characteristics of working women were examined after returning to work, and it was shown that only 32% could continue to breastfeed [8, 9]. In another study, it was shown that the total duration of breastfeeding was shorter in working mothers than in non-working mothers [10]. Women cited their return to work or school as the most common reason for stopping breastfeeding after six weeks [11].

Most mothers who returned to work after giving birth stated that they could not find the opportunity to breastfeed or express their breast milk while at work [10, 12]. As a result of a study conducted in Ghana in 2020, the importance and practices of breastfeeding and workplace factors (maternity leave, working hours, work-life support, etc.) were identified as two factors affecting breastfeeding in working mothers [13]. In another study, the necessary conditions for breastfeeding in working mothers were listed as the mother's part-time work, mother and baby not being separated for a long time, a supportive working environment and opportunities, and child care options [14].

In 2011 and 2016, changes were made to maternity leave regulations in Turkey aimed at supporting breastfeeding. These changes included extending the duration of breastfeeding leave, exempting breastfeeding mothers from night shifts, and granting the right to work part-time [15, 16]. It has been shown that these measures have a positive impact on the duration of breastfeeding among female doctors in Turkey [17]. However, there is currently no national study in Turkey evaluating the relationship between employment characteristics and the continuation of breastfeeding.

The purpose of this study was to demonstrate the relationship between mothers' breastfeeding behavior and their employment status, as well as to investigate the relationship between the mother's employment status and working conditions and the continuation of breastfeeding for infants under 24 months of age from 2008 to 2018. The study's findings will enable a more comprehensive evaluation of the factors associated with the continuation of breastfeeding in working mothers, thereby facilitating the adoption of any necessary corrective measures without compromising women's employment opportunities.

## Methods

Data for the study were obtained from the last three Turkey Demographic and Health Surveys (TDHS) conducted in 2008, 2013, and 2018. All of the TDHS were nationally representative household surveys with weighted, multistage, stratified cluster sampling collected by face-to-face interview. Sample selection was made using a similar methodology, and similar questionnaires were used in all TDHS. This allows the comparison of data from different years [18, 19].

Participants in the study comprised mothers aged 15–49 years and their children under 24 months of age, all of whom were living with their mothers. If a mother has more than one child under the age of two, the youngest children were taken. Exclusion criteria were children from multiple pregnancies and mothers who were currently pregnant. The study included 3557 (weighted 3490) child-mother pairs, from 4064 mothers aged 15–49 years, with children younger than 24 months in three TDHS.

The mother-infant couple's region (5 regions), residence (urban, rural), wealth index (lowest, second, middle, fourth, highest), the last child's characteristics [birth order, birth interval, place of delivery (home, public sector health facilities, private sector health facilities), type of birth, child age, birth weight, gender, bottle use, early initiation of breastfeeding, breast milk during the survey, whether to take], and maternal characteristics (age, education level, mother's tongue, consanguinity, employment status, characteristics of job) were taken as variables [18, 19].

The working characteristics of the mother were examined with a few questions [18, 19]: "In which month and year did you start working?" "In which sector do you work (agriculture, industry, service)?" "Do you work in the public or private sector?" "What is your status at work (employer, paid worker, etc.)?" "What is your working style in this job (full-time or part-time)?" "Were you affiliated with any social security institution while doing this job?" "If yes, which one?" "Are you still working in this job, and what was your reason for leaving? (pregnancy, marriage, child care, and so on)" Only data on the mother's full-time or part-time employment status at work was available in the TDHS 2018 [19].

Breastfed infants were defined as those who received breast milk either by nursing or by expressed breast milk during the previous day.

Bottle-fed infants were defined as those who received any liquid from a bottle with a nipple during the previous day. These infants may also be breastfed or receive expressed breast milk via bottle feeding.

The wealth index is a composite measure of a household's overall living standard. Households are given scores based on the quantity and types of consumer goods they possess, including items like televisions and cars, as well as housing attributes such as drinking water source, toilet facilities, and flooring materials. These scores are determined using principal component analysis. National wealth quintiles are constructed by assigning each usual (de jure) household member a household score, ranking individuals in the household population by their score, and dividing the distribution into five equal categories, each comprising 20% of the population [19].

### Data analyses

The IBM-SPSS 22.0 and STATA 13.0 package program was used to analyze the data. The TDHS 2008, 2013, and 2018 datasets were combined. To prevent potential biases in the analyses resulting from the different number of observations in different surveys and to perform complex sample analysis, a CSPLAN file was created using sample cluster for the primary sampling unit, sample stratum number, and sample weight. Percentage changes and 95% confidence intervals (CI) in the frequency of subgroups of each variable still breastfeeding their 0–23 month-old infants during the survey period were analyzed using complex sample crosstabs, both within the same year and between years, and Chi-square determined their significance. Adjusted residual calculations were used to determine the subgroup that created the difference in the case where more than two subgroups were compared, and significance was determined.

In all three TDHS periods, the relationship between continued breastfeeding (dependent variable) and maternal employment (never worked vs still working; worked in the past, not currently working vs still working) in 0–23 month-old infants was analyzed, with two models using the complex sample logistic regression method; Model 0: age of the child, Model 1: sociodemographic characteristics of the mother, smoking status, and child characteristics.

In all three survey periods, the relationship between the continuation of breastfeeding (dependent variable) in infants aged 0–23 months and both the sector in which the mother works (Industry vs Agriculture; Service vs Agriculture) and the characteristics of the institution (Private vs Public) (independent variables) were separately analyzed with two models by the regression method; Model 0: age of the child, Model 1: sociodemographic characteristics of the mother, smoking status, and child characteristics. For the TDHS-2018 period, the relationship between the continuation of breastfeeding (dependent variable) and the mother's working type (part-time or full-time) (independent variable) in infants aged 0–23 months was examined with the same two models, using the complex sample logistic regression method. Logistic regression analysis results were given with an adjusted odds ratio and 95% CI.

P < 0.05 was accepted as significant in all the analyses.

## Results

Table 1 shows the characteristics of mother–child pairs and the mothers' working status over the course of their lives for each TDHS period. The percentages of currently breastfed children were 69.9% in 2008 and 75.1% in 2018. Nearly half of the mothers had never worked. The percentages of still-working mothers during the survey period were 20.9% in 2008, 15.4% in 2013, and 19.4% in 2018 (Table 1).

**Table 1:** Continuity of breastfeeding according to maternal and child characteristics during working periods

	TDHS-2008		TDHS-2013		TDHS-2018		p
*Variables, p values*	Overall (*n*=1191)	Breastfed	Overall (*n*=1189)	Breastfed	Overall (*n*=1110)	Breastfed	
	%*	% [95% CI]**	%*	% [95% CI]**	%*	% [95% CI]**	
*Overall*	100	69.9	100	70.3	100	75.1	
**Region**							
*West*	36.0	67.1[59.7–73.7]^a.x^	35.1	70.3[63.2–76.5]^ab.x^	37.9	75.4[69.3–80.6]^b^	0.031
*South*	10.9	61.6[52.6–69.9]^x^	14.9	61.9[54.5–68.8]^y^	15.1	71.8[64.5–78.2]	0.085
*Central*	22.6	70.3[64.3–75.6]^x^	18.0	71.2[63.6–77.8]^xy^	17.9	72.0[63.6–79.1]	0.940
*North*	5.9	59.3[48.9–69.0]^x^	5.8	66.9[59.2–73.7]^xy^	3.9	71.8[59.0–81.7]	0.350
*East*	24.5	79.8[76.1–83.0]^y^	26.2	75.2[69.6–80.1]^x^	25.2	79.5[74.3–83.9]	0.306
* p*		0.001		0.036		0.252	
**Residence**							
*Urban*	73.0	69.3[65.3–73.1]^a^	81.0	69.3[65.3–72.9]^a^	74.7	74.9[71.1–78.3]^b^	0.012
*Rural*	27.0	71.2[65.8–76.2]	19.0	74.6[68.8–79.6]	25.3	75.9[69.8–81.1]	0.390
* p*		0.571		0.106		0.811	
**Wealth index**							
* Lowest*	20.9	79.5[74.8–83.5]^x^	19.9	75.3[69.4–80.3]	20.5	73.9[67.1–79.7]	0.335
* Second*	22.0	67.5[60.6–73.7]^a. xy^	21.5	70.5[63.7–76.4]^a^	22.1	78.7[71.8–84.3]^b^	0.015
* Middle*	22.3	70.8[64.0–76.8]^a. xy^	21.8	69.3[62.3–75.5]^a^	20.4	79.4[72.5–84.9]^b^	0.031
* Fourth*	18.0	69.6[61.7–76.5]^x^	18.5	73.0[65.7–79.2]	18.4	72.5[63.1–80.3]	0.171
* Highest*	16.8	59.9[49.2–69.8]^y^	18.4	63.2[53.8–71.8]	18.5	70.3[61.0–78.1]	0.081
* p*		0.001		0.061		0.125	
**Maternal age at birth**							
< 20* years*	7.8	64.1[53.0–73.9]	6.1	68.2[56.2–78.2]	5.3	54.8[38.8–69.9]^x^	0.310
20–34* years*	83.1	69.9[66.3–73.2]^a^	80.6	70.1[66.4–73.6]^a^	78.9	76.1[72.7–79.3]^b. y^	0.004
≥ 35* years*	9.1	74.6[62.8–83.6]	13.3	72.1[63.2–79.5]	15.9	77.0[67.8–84.2]^y^	0.576
* p*		0.266		0.801		0.001	
**Maternal education**							
* No/primary incomplete*	18.3	81.6[75.9–86.2]^x^	17.6	78.1[72.4–82.8]^x^	14.4	86.3[79.1–91.3]^x^	0.133
* Primary complete*	45.6	66.5[61.4–71.3]^y^	30.3	70.7[65.0–75.7]^y^	21.5	73.2[65.4–79.8]^y^	0.148
* Second complete*	12.5	71.6[63.2–78.7]^y^	22.4	67.5[60.0–74.2]^y^	28.9	70.0[63.1–76.1]^y^	0.661
* High complete*	23.6	66.2[59.2–72.7]^a.y^	29.7	67.4[60.8–73.5]^a.y^	35.3	76.0[69.9–81.2]^b.y^	0.008
* p*		0.001		0.037		0.001	
**Mother's tongue**							
* Turkish*	74.0	66.2[62.2–70.0]^a.x^	70.3	67.8[63.7–71.6]^a.x^	70.2	72.6[68.3–76.5]^b^	0.016
* Kurdish*	22.2	82.7[78.7–86.4]^y^	24.8	78.0[72.7–82.5]^y^	21.0	81.3[74.4–86.8]	0.346
* Others*	3.8	66.1[48.8–80.0]^x^	3.6	70.5[53.2–83.4]^xy^	8.8	80.7[71.5–87.4]	0.153
* Missing*			1.3				
* p*		0.001		0.009		0.010	
**Consanguinity**							
* Not relative*	75.4	69.5[65.6–73.2]^a^	73.6	68.7[64.9–72.3]^a.x^	78.1	76.4[72.8–79.7]^b^	0.001
* Close relative*	13.1	72.0[64.1–78.8]^a^	13.8	82.0[75.3–87.1]^b.y^	11.1	69.2[59.9–77.2]^a^	0.029
* Distant relative*	11.3	69.0[60.1–76.7]	12.4	66.6[57.7–74.6]^x^	10.6	71.7[60.3–80.9]	0.643
* Missing*	0.2		0.2		0.2		
* p*		0.730		0.005		0.216	
**Smoking**							
* Mother is a smoker*	18.4	61.6[54.1–68.5]^x^	18.6	56.2[47.9–64.1]^x^	17.8	58.6[49.3–67.3]^x^	0.504
* Non-smoking mothers with someone smoking at home*	38.7	71.7[66.8–76.2]^y^	25.0	70.4[63.9–76.2]^y^	21.1	76.7[70.2–82.1]^y^	0.241
* Non-smoking mothers with no one smoking at home*	42.9	71.7[66.6–76.3]^a.y^	56.4	74.7[70.7–78.4]^a.y^	61.1	79.4[75.4–83.0]^b.y^	0.009
* p*		0.013		0.001		0.001	
**Birth order**							
* 1*	37.0	66.3[61.1–71.2]^a.x^	29.1	64.9[59.0–70.4]^a.x^	32.4	73.7[67.0–79.5]^b^	0.023
* 2–3*	46.2	69.7[64.8–74.2]^x^	54.3	71.3[66.7–75.5]^y^	54.2	74.9[71.0–78.5]	0.126
≥ *4*	16.7	78.0[71.7–83.2]^y^	16.5	76.5[69.4–82.4]^y^	13.4	79.5[71.1–85.9]	0.837
* p*		0.011		0.014		0.435	
**Birth interval**							
* First birth*	37.0	66.3[61.1–71.2]^a^	29.1	64.9[59.0–70.4]^a.x^	32.4	73.7[67.0–79.5]^b^	0.023
< *24 mo*	10.3	72.0[63.3–79.4]	10.1	71.1[60.8–79.6]^xy^	12.7	76.6[67.4–83.9]	0.546
* 24–35 mo*	52.7	71.9[67.1–76.2]	60.8	72.7[68.9–76.3]^y^	54.9	75.6[71.5–79.3]	0.284
* p*		0.115		0.036		0.742	
**Delivery place**							
* Home*	4.1	77.9[65.4–86.8]	1.5	61.7[37.4–81.2]	0.3	–––––––-	0.180
* Public health facilities*	72.7	69.7[66.2–73.1]^a^	58.4	71.7[67.7–75.3]^a^	57.4	76.8[72.7–80.6]^b^	0.009
* Private health facilities*	22.8	69.4[61.6–76.2]	39.9	68.6[63.4–73.4]	42.0	72.5[67.1–77.4]	0.390
* Missing*	0.3	22.3[1.8–82.1]	0.2	66.4[15.1–95.6]	0.2	–––––––-	0.223
* p*		0.143		0.481		0.201	
**Delivery type**							
* Vaginal delivery*	56.4	72.5[68.6–76.0]	52.8	72.7[68.1–77.0]	45.9	77.0[72.6–80.9]	0.150
* Cesarean section*	43.6	66.5[61.1–71.4]^a^	47.2	67.6[62.7–72.2]^a^	54.1	73.6[68.8–77.8]^b^	0.020
* p*		0.026		0.056		0.186	
**Child age**							
* 0–5 months*	27.2	96.9[94.3–98.3]^x^	24.9	93.5[89.6–96.1]^x^	27.8	92.8[88.5–95.6]^x^	0.056
* 6–11 months*	25.1	78.6[72.5–83.7]^y^	27.3	76.9[70.4–82.2]^y^	27.8	83.1[77.8–87.3]^y^	0.134
* 12–17 months*	26.0	65.0[58.1–71.3]^z^	25.5	69.9[63.8–75.5]^y^	27.4	67.4[60.6–73.6]^z^	0.415
* 18–23 months*	21.7	31.6[25.2–38.7]^a.t^	22.4	36.8[36.5–43.7]^ab.z^	17.1	45.9[38.0–54.1]^b.t^	0.009
* p*		0.001		0.001		0.001	
**Sex**							
* Male*	50.6	71.9[68.3–75.3]	52.9	70.7[65.8–75.1]	46.4	74.6[69.6–79.0]	0.341
* Female*	49.4	67.7[62.4–72.6]^a^	47.1	69.9[65.3–74.1]^a^	53.6	75.7[71.2–79.7]^b^	0.008
* p*		0.115		0.799		0.728	
**Birth weight**							
< *2500 g*	7.6	56.8[45.0–67.9]^x^	7.3	60.5[47.4–72.3]	9.6	71.4[59.1–81.2]	0.097
≥ *2500 g*	83.1	69.6[65.8–73.0]^a.y^	89.6	70.7[67.1–74.1]^a^	89.0	75.6[72.0–78.8]^b^	0.007
* Don't know*	9.3	83.1[76.7–88.1]^z^	3.1	80.6[69.4–88.4]	1.4	73.7[50.4–88.6]	0.743
* p*		0.001		0.054		0.585	
**Bottle-feeding**							
* No*	50.9	86.9[83.7–89.5]^a^	47.9	86.7[83.1–89.7]^a^	49.4	94.8[92.1–96.7]^b^	0.001
* Yes*	49.1	52.1[47.5–56.7]	52.1	55.1[50.3–58.9]	50.6	55.9[50.8–61.0]	0.383
* p*		0.001		0.001		0.001	
**Early initiation of BF**							
* Within 1st hour*	50.0	73.1[68.6–77.2]^x^	65.3	71.7[67.5–75.6]^xy^	71.7	75.2[71.2–78.8]^x^	0.275
* 1st-* < *2nd hour*	18.8	70.7[63.0–77.3]^x^	11.4	79.3[71.1–85.7]^y^	7.1	78.1[65.4–87.0]^x^	0.141
≥ *2nd hour*	29.5	67.8[61.8–73.3]^a.y^	21.7	66.8[60.1–72.8]^a.x^	19.7	79.7[72.7–85.3]^b.y^	0.002
* Don't know*	1.7		1.7		1.5		
* p*		0.001		0.001		0.001	
**Current working status of the mother**							
* Worked in the past, not currently working*	33.3	73.4[67.7–78.5]^x^	37.8	69.5[64.6–73.9]	35.4	73.8[67.9–78.9]	0.301
* Currently working*	20.9	59.5[52.3–66.4] ^a.y^	15.4	67.3[59.2–74.5]^a^	19.4	78.8[71.3–84.8]^b^	0.001
* Never worked*	45.8	72.0[67.7–75.9]^x^	46.8	71.9[67.4–71.6]	45.1	74.7[69.6–79.1]	0.533
* p*		0.001		0.429		0.384	

^*^Weighted sample, column percentages, **Weighted sample, row percentages, 95% confidence interval

^xyzt^Values with different letters in the same column are different, *p* < 0.05; ^ab^Values with different letters in the same line are different, *p* < 0.05

Looking at the work sectors of the mothers, the change over the years was quite similar both in the past and current employment sectors (Supplementary Table 1). While the agricultural sector declined, the service sector increased. The percentages of mothers still working in the public sector were 23.3% in 2008 and 36.6% in 2018.

Among the reasons for mothers to leave their last job, the highest ratio was for marriage. It was 40.6% in 2008 and 36.5% in 2018. The second reason was “got pregnant/childcare” (20.5% in 2008, 25.5% in 2013, 26.5% in 2018; Table 2).

**Table 2:** Reasons for leaving the job of previously employed women according to TDHS periods, %*

**Reason for resignment from last work**	TDHS-2008 (*n* = 397)	TDHS-2013 (*n* = 449)	TDHS-2018 (*n* = 393)
Marriage	40.6	32.3	36.5
Got pregnant/childcare	20.5	25.5	26.5
Moved/migrated	4.1	2.3	3.7
The opposition of partner/elderly	3.4	4.1	2.4
Workplace closed	2.0	2.7	2.7
Fired	1.4	2.9	2.8
To find/found a better job	0.3	1.0	0.8
Problems at workplace	2.7	7.2	3.2
Seasonal/temporary	0.9	1.8	4.2
Sick/elderly care in family	3.6	0.4	0.6
Sick/disabled	1.2	4.6	1.9
Retirement	0.2	–	–
Did not need to work	1.8	2.3	0.2
Did not want to work	11.1	9.4	5.9
Education	–	0.7	–
Other	16.3	2.8	8.5
*Weighted sample, column percentages			

Overall, the East region had a significantly higher percentage of breastfeeding than other regions in 2008 (79.8%). Compared to 2008, the breastfeeding percentage in the West region was found to be higher in 2018 (67.1% vs 75.4%, p = 0.031; Table 1). It was observed that breastfeeding prevalence varies according to the mother's spoken language, with the highest prevalence found among Kurdish speakers. While there were no changes observed in breastfeeding prevalence among Kurdish speakers across three periods, an increase in breastfeeding prevalence was noted among Turkish speakers.

It was observed that breastfeeding prevalence among mothers under the age of 20 was significantly lower in 2018 compared to mothers aged 20 and above (Table 1). Considering the breastfeeding prevalence according to maternal education levels, the highest percentage was among mothers who did not receive any education in all surveys. Breastfeeding percentages increased significantly among the first deliveries, infants born by cesarean section, and infants born in public health facilities from TDHS-2008 and TDHS-2013 to TDHS-2018 (p = 0.023, p = 0.020, and p = 0.009, respectively; Table 1). In 2018, breastfeeding prevalence was higher compared to previous years, significantly notable in cases of Cesarean section (CS) births.

The age of the infants was significant in terms of breastfeeding. The highest breastfeeding percentages were in 0–5 month infants (96.9% in 2008, 93.5% in 2013, and 92.8% in 2018). However, it increased in infants aged 18–23 months from 2008 to2018. Breastfeeding prevalences among mothers who practice mixed feeding and provide other foods to their infants through bottle feeding were significantly lower than in their peers (52.1% vs. 86.9% in 2008, 55.1% vs 86.7% in 2013, and 55.9% vs 94.8% in 2018; Table 1).

**Fig. 1:**
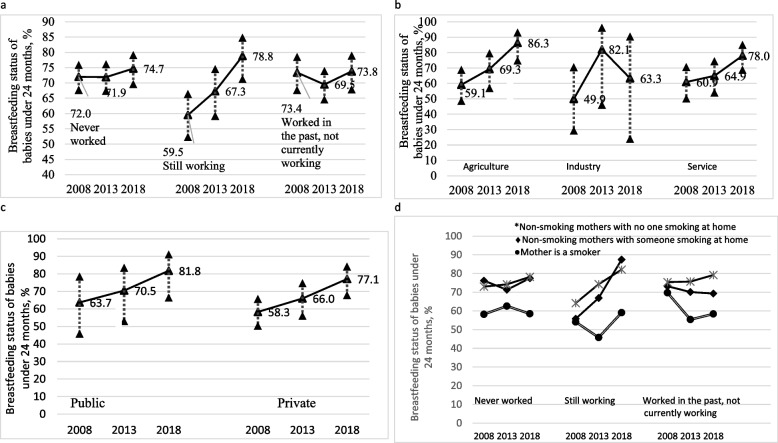
**a** The change in breastfeeding over the years with the mother's work. **b** Change in breastfeeding status over the years according to the last sector of the mother. **c** Change of breastfeeding over the years according to the mother's employment in the public-private sector. **d** Changes in maternal work and the effect of smoking on breastfeeding over the years

Breastfeeding prevalences of actively working mothers in 2008 were significantly lower than those of mothers who had never worked or who had worked for a period of their lives and then quit (p < 0.001). This difference was not observed in other survey years, (Fig. 1, Table 1). It is observed that breastfeeding prevalence significantly increased among employed women from 2008 through 2018, while there was no change in breastfeeding prevalence among women who left their jobs or had never been employed before (Table 1).

Breastfeeding percentages were the lowest in women whose mothers smoked in all periods (61.6% in 2008, 56.2% in 2013, and 58.6% in 2018, Table 1). The relationship between the mother's work and smoking varied according to year (Fig. 1).

The breastfeeding percentages for mothers working in agriculture and those working in the service sector were significantly higher in 2018 compared to previous years (*p* = 0.025 for agriculture, *p* = 0.018 for service sector). While it increased in 2013 compared to 2008 industry workers, it decreased again in 2018 (Fig. 1, Table 3). The breastfeeding percentage of mothers working in the private sector was significantly higher in 2018 compared to previous years (*p* = 0.026, Fig. 1).

**Table 3:** Continuing breastfeeding status of mothers by employment characteristics and according to workplace characteristics

	TDHS-2008		TDHS-2013		TDHS-2018		p
	Overall (*n* = 246)	Breastfed	Overall *(n* = 183)	Breastfed	Overall (*n* = 216)	Breastfed	
	%*	% [95% CI]**	%*	% [95% CI]**	%*	% [95% CI]**	
**Sector of work**							
* Agriculture*	37.3	59.1[48.6–68.8]^a^	30.1	69.3[56.9–79.5]^a^	18.7	86.3[74.8–93.0]^b^	0.025
* Industry*	6.2	49.9[29.3–70.5]	6.4	82.1[46.1–96.1]	4.9	63.3[24.0–90.4]	0.839
* Service*	56.4	60.9[50.2–70.6]^a^	63.5	64.9[54.0–74.4]^a^	76.4	78.0[68.8–85.0]^b^	0.018
* p*		0.693		0.397		0.271	
**Public/private**							
Public	23.3	63.7[45.9–78.5]	28.8	70.5[53.1–83.5]	36.6	81.8[66.5–91.1]	0.180
* Private*	76.7	58.3[50.4–65.7]^a^	71.2	66.0[56.1–74.7]^a^	63.4	77.1[66.5–91.1]^b^	0.026
* p*		0.542		0.729		0.487	
**Status of work**							
* Employer*	3.2	42.7[10.6–82.4]^xy^	2.9	57.9[25.0–85.1]	1.2	………….	0.077
* Waged worker (regular)*	22.9	70.6[63.8–76.7]^xy^	18.8	69.4[62.6–75.4]	32.0	72.2[66.1–77.6]	0.276
* The salaried government official*	19.3	66.6[49.1–80.4]^xy^	28.0	68.1[48.7–82.7]	33.4	77.4[63.2–87.2]	0.403
* Daily waged (seasonal/temporal)*	8.3	81.5[71.5–88.5]^y^	9.1	71.9[59.9–81.5]	6.0	88.4[74.4–95.2]	0.360
* For her own (regular)*	3.7	38.7[13.6–71.6]^x^	8.4	80.9[38.7–96.6]	3.6	61.9[29.2–86.4]	0.006
* For her own (irregular)*	11.4	68.3[51.5–81.3]^xy^	9.6	62.8[43.4–78.7]	8.2	75.0[51.5–89.4]	0.221
* Unpaid family worker*	31.1	61.8[53.8–69.3]^x^	23.2	69.1[58.7–77.8]	14.7	86.1[74.4–93.0]	0.002
* p*		0.017		0.670		0.078	
**Full/part-time**							
* Full-time*					81.4	77.3[68.7–84.0]	
* Part-time*					18.6	85.7[70.9–93.6]	
* p*						0.392	
**Social security for work**							
* None*	52.1	67.8[62.6–72.6]	48.8	69.0[63.6–73.9]	32.4	77.8[71.7–82.9]	0.360
* Public*	47.8	68.6[61.3–75.2]	50.8	69.4[61.2–76.4]	66.8	73.7[67.2–79.4]	0.054
* Private insurance/Other*	0.2	29.1[3.5–82.1]	0.3	42.9[11.5–81.4]	0.8	53.9[6.8–94.9]	
* p*		0.337		0.394		0.394	

^*^Weighted sample, column percentages

^**^Weighted sample, row percentages, 95% confidence interval

^ab^Values with different letters in the same row are different, ^x,y^Values with different letters in the same column are different, *p* < 0.05

When the change according to years is analyzed by modeling, the percentage of breastfeeding in mothers who have never worked in model 0 in 2008 was 1.47 times higher (95% CI: 1.00–2.16) compared to working mothers. In Model 1, this significance disappeared when confounding factors were controlled. In 2013 and 2018, maternal employment status did not affect breastfeeding significantly in either model 0 or model 1 (Table 4).

**Table 4:** Multivariate logistic regression analysis of the association of mother-child variables with continued breastfeeding and the relationship between continued breastfeeding and the mother's industry, institution, and full-time employment in children aged 0-23 months over three TDHS periods (Model: Enter), AOR (95% CI)

	TDHS-2008	TDHS -2013	TDHS -2018
Mother's working status			
Model 0*			
*Never worked vs Still working*	**1.47 (1.00-2.16)**	1.12 (0.69-1.81)	0.72 (0.43-1.20)
*Worked in the past, not currently working vs Still working*	1.52 (0.95-2.43)	0.98 (0.60-1.60)	0.71 (0.42-1.21)
Model 1**			
*Never worked vs Still working*	1.15 (0.76-1.73)	0.86 (0.51-1.46)	0.70 (0.38-1.31)
*Worked in the past, not currently working vs Still working*	1.53 (0.92-2.52)	0.95 (0.56-1.61)	0.67 (0.38-1.17)
**The sector of current work**			
Model 0*			
*Industry vs Agriculture *	0.58 (0.28-1.23)	1.55 (0.42-5.76)	**0.20 (0.05-0.85)**
*Service vs Agriculture*	1.25 (0.67-2.34)	0.58 (0.28-1.22)	0.59 (0.27-1.28)
Model 1**			
*Industry vs Agriculture*	0.28 (0.07-1.04)	1.99 (0.27-14.52)	**0.06 (0.01-0.48)**
*Service vs Agriculture*	0.52 (0.14-1.90)	0.68 (0.15-3.05)	0.34 (0.09-1.24)
**Public/private current work**			
Model 0*			
*Private vs Public*	0.59 (0.29-1.23)	1.29 (0.59-2.85)	1.04 (0.54-2.00)
Model 1**			
*Private vs Public*	0.72 (0.33-1.57)	0.79 (0.33-1.89)	0.95 (0.44-2.06)
**Full/part-time current work**			
Model 0*			
*Part vs full-time*			**1.93 (1.42-2.61)**
Model 1**			
*Part vs full-time*			1.15 (0.65-2.03)

*AOR *Adjusted odds ratio, *CI *Confidence interval

^*^The age of the child has been included

^**^Region, residence, wealth index, maternal age at birth, maternal education, mother's tongue, consanguinity, number of siblings, place of birth, mode of birth, gender, birth weight, early initiation of breastfeeding, and smoking characteristics were taken as confounding factors

In 2008 and 2013, there was no relationship between the sector in which the mother worked and breastfeeding in model 0 and model 1. In 2018, mothers working in the industry had a lower breastfeeding odds (AOR: 0.20, 95% CI: 0.05–0.85) than mothers working in agriculture in model 0. In Model 1, the significance was remained. No interactions were detected between public/private work and breastfeeding status. Mother's full-time/part-time employment information was available only in 2018. In further analysis, mothers who worked part-time in model 0 had higher odds for breastfeeding (AOR: 1.93, 95% CI: 1.42–2.61) compared to full-time workers. However, the relationship disappeared when confounding factors were adjusted in Model 1 (Table 4).

The median duration of breastfeeding has increased over the years (17.4 months in 2008, 19.0 months in 2013, and 20.2 months in 2018). Taking employment status into account, the median duration of breastfeeding has increased over time, both in women who have never worked and in women who have worked. The median duration of breastfeeding in currently working mothers has also increased over the years (16.2 months in 2008, 20.0 months in 2013, and 21.8 months in 2018). In mothers who had worked before but are not currently working, the median duration of breastfeeding increased to 18.1 months in 2008, 19.2 months in 2013, and decreased to 16.7 months in 2018 (Table 5). According to the sector in which the mother works, an increase was observed in mothers working in agriculture from 2008 to 2013, while this increase was not observed in 2018 (17.0 months in 2008, 20.8 months in 2013, and 20.5 months in 2018). Similar to agriculture, the median duration of breastfeeding increased from 2008 to 2013 among service sector mothers, while it was similar to 2013 in 2018 (17.6 months in 2008, 19.2 months in 2013, and 19.0 months in 2018). Although the mean duration of breastfeeding among mothers working in the industry increased from 2008 to 2013, it decreased in 2018. (16.4 months in 2008, 19.0 months in 2013, 15.8 months in 2018).

**Table 5:** Median duration of breastfeeding, month

	TDHS-2008	TDHS-2013	TDHS-2018
Overall	17.4	19.0	20.2
Never worked	17.6	18.1	19.9
Still work, No	18.1	19.2	16.7
Still work, Yes	16.2	20.0	21.8
Sector, agriculture	17.0	20.8	20.5
Sector, industry	16.4	19.0	15.8
Sector, service	17.6	19.2	19.0

## Discussion

Various factors were identified as influencing the continuation of breastfeeding, including the region of residence, welfare level, mother's age, education level, mother's tongue, child's birth order, birth type, child's age, birth weight, bottle use, early initiation of breastfeeding, and smoking, all demonstrating statistically significant differences. The variation in breastfeeding prevalence according to the mother's spoken language indicates differences in breastfeeding practices based on cultural and ethnic backgrounds [12, 20]. The statistical significance of higher breastfeeding prevalence among CS births in the 2018 DHS study, and its approximation to the value observed in vaginal births, may be associated with the effectiveness of Baby-Friendly Hospital Initiative practices [21, 22].

In our study, we found that the breastfeeding prevalence is higher among mothers with lower education levels. There are conflicting reports on the relationship between maternal education and the continuation of breastfeeding [10, 12, 23, 24]. In studies conducted in Turkey and among immigrants, it has been reported that the percentage of continued breastfeeding decreases as the mother's level of education increases [12, 24]. This can be explained by the prevalence of traditional practices being more common among those with lower education levels. Additionally, this may be related to the mother's increased ability to afford formula as her education level rises, possibly without fully understanding the importance of breast milk and breastfeeding, and assuming that those with lower income have no other options due to the cost.

While maternal employment held significance in terms of breastfeeding in the TDHS 2008, this significance did not persist in subsequent years and the overall dataset. The specific working characteristics of the mother, such as the sector of employment (public or private), work status, and social security, did not exhibit a significant impact on breastfeeding.

Interestingly, although breastfeeding prevalences were initially higher among mothers who had never worked compared to working mothers, this difference disappeared after controlling for confounding factors. This increase is attributed to a better understanding of breastfeeding's importance among mothers, favorable working conditions for breastfeeding, and supportive government and workplace policies promoting breastfeeding. A study by Kang et al. [25], among Korean women, found that breastfeeding initiation percentages were similar regardless of maternal working status. However, the percentages of sustained breastfeeding decreased over time in both working and non-working mothers, with a more notable decline observed in working mothers.

In our study, while the mother's work was significant regarding breastfeeding in TDHS 2008, it was not significant in the following years or the available total. It is thought that this change may be related to the policies to support breastfeeding in our country and the change in the law regulating maternity leave in 2011. In a study conducted in our country, the breastfeeding experiences of female physicians were examined along with the effects of the regulation made in 2011 on working hours after maternity leave. It was observed that the percentages of exclusive breastfeeding and continued breastfeeding for more than 12 months increased significantly after the new law [17]. In Turkey, civil servant mothers have 16 weeks of paid maternity leave after giving birth. After this leave, mothers have the right to breastfeed for 1 year, 3 h a day in the first 6 months, and 1.5 h a day in the second 6 months. In addition, civil servant mothers cannot be assigned to the night shift for 2 years after the 24th week of pregnancy. Mothers have the option to extend their time off after the standard postpartum maternity leave period by utilizing unpaid leave for upto 24 months [15, 16]. While working, mothers have a total of 16 weeks of maternity leave, 8 weeks before birth and 8 weeks after birth, and a total of 1.5 h of breastfeeding leave per day to breastfeed their children under the age of one. If they wish, they can take up to 6 months of unpaid leave after the completion of the 16-week period. In addition, depending on their wishes, unpaid leave can be given for sixty days in the first birth, one hundred and twenty days in the second birth, and one hundred and eighty days in the following births, which is half of the weekly working time [26]. "The World Health Organization (WHO), the International Labor Organization (ILO), and the World Alliance for Breastfeeding Action advocate for employers to adhere to national laws governing paid maternity leave, breastfeeding breaks, and breastfeeding support [27, 28]. They have outlined specific guidelines, including the provision of facilities such as dedicated rooms or nurseries for children, flexible working hours, and the arrangement of suitable intervals for mothers to return to work. The ILO particularly recommends a minimum of 14 weeks of paid maternity leave and a reduction in daily working hours to facilitate breastfeeding. Additionally, it advises the establishment of breastfeeding and expressing facilities with proper hygiene conditions either at the workplace or in close proximity [29]. UNICEF's latest family-friendly policy briefing recommends at least six months of paid leave to include both parents, with 18 weeks reserved for the mother [30]. However, around 60% of working women worldwide make a living in the informal economy, and as a result, this population of mothers does not enjoy formal employment-related protections known to improve infant and young child feeding practices [31, 32]. According to ILO data, more than 90% of women in South Asia, 92% in sub-Saharan Africa, and 54.3% in Latin America and the Caribbean are employed in the informal economy [33]. It is necessary to support informal working mothers to maintain their livelihood, protect their own health, and feed their children. Due to their limited legal protection, these mothers need more social support than formal workers.

In a study conducted in the USA in 2021, mothers who did not work in the first 6 months, mothers in professional occupations, and mothers working in the service sector were compared, and the latter were found to have the shortest breastfeeding period among the three groups. In the same study, no significant difference was found between non-working mothers and mothers with professional occupations in terms of breastfeeding duration [34]. In our study, the breastfeeding percentages of mothers working in the agriculture, service, and industry sectors were not statistically different from each other in univariate analysis. Looking at the change over the years, breastfeeding percentages of mothers and the median duration of breastfeeding in the agriculture and service sectors have increased over the years. In 2018, a significant increase was detected in both sectors compared to previous years. It is thought that the high prevalence of breastfeeding among mothers working in agriculture in our country may be due to the fact that the mother-baby pair can be together in the working environment, and that the mother can frequently breastfeed because she can work in close proximity to her baby. Breastfeeding percentages of mothers working in industry were found to be significantly lower than those of mothers working in agriculture in a subsequent analysis of our study. For this reason, it is thought that policies supporting breastfeeding should be developed for mothers working in the industry in our country.

In the present study, there was no significant difference in breastfeeding percentages among mothers working in the public and private sectors in both univariate and multivariate analyses. In a study conducted in Kenya in 2002, it was found that the breastfeeding duration of women working in the private sector was shorter than that of those working in the public sector [35]. As we analyze changes across various sectors, it's clear that there hasn't been a significant shift in breastfeeding prevalences among mothers employed in the public sector from 2008 to 2018. In contrast, breastfeeding prevalences among mothers working in the private sector have shown a consistent increase over time. In 2018, it was found to be significantly higher than in previous years. It is thought that the increase in breastfeeding percentages of mothers working in the private sector may be related to the increased awareness of both mothers and employers about the importance of breast milk with the initiative of "Baby-Friendly Cities" [21].

The conditions necessary for the continuation of breastfeeding in working mothers are listed as the mother's part-time work, mother and baby not being separated for a long time, a supportive working environment and opportunities, and child care options [14]. In our study, there is only 2018 data about the mother's full-time or part-time work. The data in 2018 revealed that working full-time or part-time did not make a statistically significant difference in terms of breastfeeding duration. In further analysis, no significance was detected even after removing confounding factors. The part-time working model is not very common in our country. This result was thought to be related to the low number of cases.

### Strengths and limitations

Our study has some limitations. Because of the cross-sectional design of TDHS data, it should be noted that the factors identified do not show causation. Since the data were obtained by asking the mothers, there may be biases due to inaccurate or incomplete recall. There may be other variables that are unknown and not included in the study. The TDHS does not include questions such as workplace conditions (milking room, pump, refrigerator, etc.) and employers' and colleagues' attitudes toward breastfeeding. TDHS does not include a qualitative methodology. Therefore, some known factors that may be related to the work of mothers, such as some social beliefs, cultural practices, and education during pregnancy, were not included in the analysis [20, 24]. Also, the lack of analysis on the mother's marital status and the employment status of her spouse is also a limitation [36]. Additional studies, including qualitative components, will reveal the relationship between maternal work and breastfeeding more clearly.

In the TDHS, international standard questionnaires are used to provide time variation within the country and comparison with other countries. On the other hand, the study has the power to be a nationally representative study. Further analyses were also carried out to explain the sampling strategy and sample weight, so our findings are generalizable to the whole country.

## Conclusions

Our study showed that the mother's job did not prevent the continuation of breastfeeding. It was seen that policies should be developed for mothers working in the industry in order to continue breastfeeding while protecting women's employment. It is observed that over one-third of previously employed women leave their jobs due to marriage, and one-fourth due to pregnancy. However, it was noted that there was no statistically significant difference in breastfeeding prevalences among employed women compared to those who are homemakers in 2013 and 2018 data. Informing mothers that breastfeeding can effectively continue while working may reduce the rate of job resignation due to these reasons and support the continuity of female employment. Pediatrists can play a vital role by offering counseling and social support aimed at enhancing the health of both mothers and children [37]. They can also collaborate with institutions to implement social and economic frameworks that support breastfeeding among working mothers, thus helping to maintain maternal employment. Breastfeeding policies and practices differ across countries and industries. Therefore, conducting international research to gain insight into the experiences of women who leave their jobs due to marriage or breastfeeding is essential. Such research can greatly aid policymakers and healthcare professionals in offering improved support and allocating necessary resources.

### Supplementary Information

Supplementary Material 1: Supplementary Table 1. Job characteristics of mothers who have ever worked at any point in their lives and during the survey application period, according to TDHS periods*

## Data Availability

The data of this study are available from the Hacettepe University Institute of Population Studies. To get authorization (1) New User Registration and (2) Provide information on your study are necessary via https://tnsaveri_tdhsdata.hacettepe.edu.tr/request.php.

## References

[1] Gartner LM, Morton J, Lawrence RA, et al. Breastfeeding and the use of human milk. Pediatrics. 2005;115(2):496–506. 15687461 10.1542/peds.2004-2491

[2] Del Ciampo LA, Del Ciampo IRL. Breastfeeding and the benefits of lactation for women’s health. Rev Bras Ginecol Obstet. 2018;40(6):354–9. 29980160 10.1055/s-0038-1657766 PMC10798271

[3] Binns C, Lee M, Low WY. The long-term public health benefits of breastfeeding. Asia Pac J Public Health. 2016;28(1):7–14. 26792873 10.1177/1010539515624964

[4] Piro E, Serra G, Schierz IAM, Giuffrè M, Corsello G. Fetal growth restriction: a growth pattern with fetal, neonatal and long-term consequences. Euromediterranean Biomedical Journal. 2019;14(09):038–44.

[5] WHO. Infant and young child feeding: a tool for assessing national practices, policies and programmes. Geneva: World Health Organization; 2003.

[6] Snyder K, Hansen K, Brown S, Portratz A, White K, Dinkel D. Workplace breastfeeding support varies by employment type: the service workplace disadvantage. Breastfeed Med. 2018;13(1):23–7. 29185806 10.1089/bfm.2017.0074

[7] Ogbuanu C, Glover S, Probst J, Hussey J, Liu J. Balancing work and family: effect of employment characteristics on breastfeeding. J Hum Lact. 2011;27(3):225–38; quiz 293-225. 21393503 10.1177/0890334410394860

[8] Dun-Dery EJ, Laar AK. Exclusive breastfeeding among city-dwelling professional working mothers in Ghana. Int Breastfeed J. 2016;11:23. 27602050 10.1186/s13006-016-0083-8 PMC5012076

[9] Bai DL, Fong DYT, Tarrant M. Factors associated with breastfeeding duration and exclusivity in mothers returning to paid employment postpartum. Matern Child Health J. 2015;19(5):990–9. 25095769 10.1007/s10995-014-1596-7

[10] Bertini G, Perugi S, Dani C, Pezzati M, Tronchin M, Rubaltelli FF. Maternal education and the incidence and duration of breast feeding: a prospective study. J Pediatr Gastroenterol Nutr. 2003;37(4):447–52. 14508215 10.1097/00005176-200310000-00009

[11] Brown CR, Dodds L, Legge A, Bryanton J, Semenic S. Factors influencing the reasons why mothers stop breastfeeding. Can J Public Health. 2014;105(3):e179–85. 25165836 10.17269/cjph.105.4244 PMC6972160

[12] Yalçin SS, Erat Nergiz M, Elci ÖC, et al. Breastfeeding practices among Syrian refugees in Turkey. Int Breastfeed J. 2022;17:10. 35164812 10.1186/s13006-022-00450-3 PMC8842938

[13] Abekah-Nkrumah GAM, Nkrumah J, Gbagbo FY. Examining working mothers’ experience of exclusive breastfeeding in Ghana. Int Breastfeed J. 2020;17:56. 10.1186/s13006-020-00300-0 PMC7302356 32552899

[14] Johnston ML, Esposito N. Barriers and facilitators for breastfeeding among working women in the United States. J Obstet Gynecol Neonatal Nurs. 2007;36(1):9–20. 17238942 10.1111/j.1552-6909.2006.00109.x

[15] Devlet Personel Başkanlığı [State Personnel Department]. Kamu personeli genel tebliği (Seri No: 2). [General communique on public staff]. Resmi Gazete 15/04/2011–27906. 2011. http://www.resmigazete.gov.tr/eskiler/2011/04/20110415-17.htm. Accessed 5 May 2022.

[16] Çalışma ve Sosyal Güvenlik Bakanlığı Devlet Personel Başkanlığı [Ministry of Labor and Social Security State Personnel Department]. Kamu personeli genel tebliği [General communique on public staff] (Seri no:6). Resmi Gazete [03/04/2016 Sayı : 29683]. 2016. https://www.resmigazete.gov.tr/Eskiler/2016/04/20160413-10.htm. Accessed 5 May 2022.

[17] Eren T, Kural B, Yetim A, Boran P, Gökçay G. Breastfeeding experiences of female physicians and the impact of the law change on breastfeeding. Turk Pediatri Ars. 2018;53(4):238–44. 30872926 10.5152/TurkPediatriArs.2017.6497 PMC6408189

[18] Hacettepe University Institute of Population Studies. 2008 Turkey demographic and health survey. Ankara: Hacettepe University Institute of Population Studies, Ministry of Health General Directorate of Mother and Child Health and Family Planning, TR Prime Ministry Undersecretary of State Planning Organization and TÜBİTAK; 2009.

[19] Hacettepe University Institute of Population Studies. 2018 Turkey Demographic and Health Survey. Ankara: Hacettepe University Institute of Population Studies, T.R. Presidency of Turkey Directorate of Strategy and Budget and TÜBİTAK; 2019.

[20] Yalçın SS, Erat Nergiz M, Yalçın S. Evaluation of breastfeeding and infant feeding attitudes among syrian refugees in Turkey: observations of Syrian healthcare workers. Int Breastfeed J. 2023;18:38. 37559070 10.1186/s13006-023-00579-9 PMC10413606

[21] Erkul PE, Yalçin SS, Kiliç S. Evaluation of breastfeeding in a Baby-friendly City, Corum, Turkey. Cent Eur J Public Health. 2010;18(1):31–7. 20586228 10.21101/cejph.a3552

[22] Çaylan N, Kiliç M, Yalçin S, Tezel B, Kara F. Baby-friendly hospitals in Turkey: evaluation of adherence to the ten steps to successful breastfeeding. East Mediterr Health J. 2022;28(5):352–61. 35670440 10.26719/emhj.22.021

[23] Yalçin SS, Berde AS, Yalçin S. Determinants of exclusive breast feeding in sub-Saharan Africa: a multilevel approach. Paediatr Perinat Epidemiol. 2016;30(5):439–49. 27259184 10.1111/ppe.12305

[24] Yalçın SS, Yalçın S, Kurtuluş-Yiğit E. Determinants of continued breastfeeding beyond 12 months in Turkey: secondary data analysis of the demographic and health survey. Turk J Pediatr. 2014;56(6):581–91. 26388587

[25] Kang NM, Lee JE, Bai Y, Van Achterberg T, Hyun T. Breastfeeding initiation and continuation by employment status among Korean women. J Korean Acad Nurs. 2015;45(2):306–13. 25947192 10.4040/jkan.2015.45.2.306

[26] Tuaç P. Çalışan Annelerin Hukuki Olarak Sahip Oldukları Haklar: Hamilelik, Lohusalık ve Analık Dönemlerine İlişkin Yasal Düzenlemeler. In: Dalkılıç Süregevil O, editors. Çalışanne Kadın Akademisyenlerin Kaleminden Çalışma Yaşamında Annelik. Ankara: Nobel Akademik Yayıncılık; 2015. p. 111–148.

[27] Addati L, Cassirer N, Gilchrist K. Maternity and paternity at work: law and practice across the world. 2014. https://www.ilo.org/wcmsp5/groups/public/@dgreports/@dcomm/@publ/documents/publication/wcms_242615.pdf.

[28] Alison L, Virginia Y. Maternity legislation: protecting women's rights to breastfeed. WABA activity sheet 6. https://waba.org.my/resources/activitysheet/acsh6.htm. Accessed 5 May 2022.

[29] Soomro JA, Shaikh ZN, Saheer TB, Bijarani SA. Employers’ perspective of workplace breastfeeding support in Karachi, Pakistan: a cross-sectional study. Int Breastfeed J. 2016;11:24. 27606000 10.1186/s13006-016-0084-7 PMC5013577

[30] UNICEF. Global breastfeeding scorecard; UNICEF policy brief on family-friendly policies 2019. https://www.unicef.org/breastfeeding/. Accessed 5 May 2022.

[31] Bhan G, Surie A, Horwood C, et al. Informal work and maternal and child health: a blind spot in public health and research. Bull World Health Organ. 2020 98(3):219–21. 10.2471/BLT.19.231258. 32132757 10.2471/BLT.19.231258 PMC7047022

[32] Moussié R, Alfers L. Pandemic, informality and women’s work: Redefining social protection priorities at WIEGO. Global Social Policy. 2022;22(1):190–5. 10.1177/14680181221079089

[33] Bonnet F, Vanek J, Chen M. Women and men in the informal economy: a statistical brief. Manchester: WIEGO; 2019. https://www.ilo.org/wcmsp5/groups/public/---ed_protect/---protrav/---travail/documents/publication/wcms_711798.pdf.

[34] Whitley MD, Ro A, Palma A. Work, race and breastfeeding outcomes for mothers in the United States. PLoS One. 2021;16(5): e0251125. 33951094 10.1371/journal.pone.0251125 PMC8099119

[35] Lakati A, Binns C, Stevenson M. The effect of work status on exclusive breastfeeding in Nairobi. Asia Pac J Public Health. 2002;14(2):85–90. 12862412 10.1177/101053950201400206

[36] Serra G, Miceli V, Albano S, Corsello G. Perinatal and newborn care in a two years retrospective study in a first level peripheral hospital in Sicily (Italy). Ital J Pediatr. 2019;45:152. 10.1186/s13052-019-0751-6. 31783883 10.1186/s13052-019-0751-6 PMC6884854

[37] Serra G, Giuffrè M, Piro E, Corsello G. The social role of pediatrics in the past and present times. Ital J Pediatr. 2021;47:239. 10.1186/s13052-021-01190-6. 34922600 10.1186/s13052-021-01190-6 PMC8684095

