# Supplementary material for: The interplay between maternal employment dynamics and trends in breastfeeding practices over the past decade
Source: Ital J Pediatr. 2024 Aug 9;50:146. doi: 10.1186/s13052-024-01697-8 (PMC11312228; doi:10.1186/s13052-024-01697-8)
Supplement: Supplementary file 1 — Supplementary Material 1: Supplementary Table 1. Job characteristics of mothers who have ever worked at any point in their lives and during the survey application period, according to TDHS periods* [file 13052_2024_1697_MOESM1_ESM.docx]

Supplementary Table 1. Job characteristics of mothers who have ever worked at any point in their lives and during the survey application period, according to TDHS periods*

|  | **Characteristics of the last job held by mothers who have ever worked** | | | **Job characteristics of currently working mothers** | | |
| --- | --- | --- | --- | --- | --- | --- |
|  | TDHS -  2008  (n=646) | TDHS -  2013  (n=632) | TDHS -  2018  (n=609) | TDHS -2008  (n=249) | TDHS -2013  (n=183) | TDHS -2018  (n=216) |
| **Sector of work** |  |  |  |  |  |  |
| *Agriculture* | 34.0 | 24.2 | 16.4 | 37.3 | 30.1 | 18.7 |
| *Industry* | 21.1 | 17.0 | 11.2 | 6.3 | 6.4 | 4.9 |
| *Service* | 44.9 | 58.8 | 72.4 | 56.4 | 63.5 | 76.4 |
| **Public/private work** |  |  |  |  |  |  |
| *Public* | 11.2 | 12.3 | 18.0 | 23.3 | 28.8 | 36.6 |
| *Private* | 88.8 | 87.7 | 82.0 | 76.7 | 71.2 | 63.4 |
| **Status of work** |  |  |  |  |  |  |
| *Employer* | 1.2 | 2.0 | 0.6 | 3.2 | 2.9 | 1.2 |
| *Waged worker(regular)* | 45.9 | 52.6 | 59.0 | 22.9 | 18.8 | 32.0 |
| *The salaried, government official* | 7.7 | 9.4 | 13.9 | 19.3 | 28.0 | 33.4 |
| *Daily waged (seasonal/temporal)* | 10.3 | 11.0 | 8.2 | 8.3 | 9.1 | 6.0 |
| *For her own (regular)* | 1.8 | 2.8 | 3.2 | 3.7 | 8.4 | 3.6 |
| *For her own (irregular)* | 6.4 | 6.2 | 6.0 | 11.4 | 9.6 | 8.2 |
| *Unpaid family worker* | 26.3 | 15.5 | 8.9 | 31.1 | 23.2 | 14.7 |
| *Other* | 0.4 | 0.5 | 0.3 | ---- | ---- | 0.8 |
| **Full-time/part-time work** |  |  |  |  |  |  |
| *Full-time* | -------- | ------------ | 86.4 | ---- | ---- | 81.4 |
| *Part-time* | --------- | ------------ | 13.6 | ---- | ---- | 18.6 |
| **Social security for work** |  |  |  | --- |  |  |
| *None* | 59.3 | 57.5 | 46.5 | 52.1 | 48.8 | 32.4 |
| *Public institution* | 40.5 | 40.8 | 52.7 | 47.8 | 50.8 | 66.8 |
| *Private insurance* | ------ | 0.2 | 0.6 | --- | ----- | 0.8 |
| *Other* | 0.2 | 1.4 | 0.2 | 0.2 | 0.3 | --- |
| *Weighted sample, column percentages | | | |  |  |  |
